# Clinical Characteristics and Prognostic Factors for Intensive Care Unit Admission of Patients With COVID-19: Retrospective Study Using Machine Learning and Natural Language Processing

**DOI:** 10.2196/21801

**Published:** 2020-10-28

**Authors:** Jose Luis Izquierdo, Julio Ancochea, Savana COVID-19 Research Group, Joan B Soriano

**Affiliations:** 1 Hospital Universitario de Guadalajara Guadalajara Spain; 2 Hospital Universitario de La Princesa Madrid Spain; 3 Savana Madrid Spain

**Keywords:** artificial intelligence, big data, COVID-19, electronic health records, tachypnea, SARS-CoV-2, predictive model

## Abstract

**Background:**

Many factors involved in the onset and clinical course of the ongoing COVID-19 pandemic are still unknown. Although big data analytics and artificial intelligence are widely used in the realms of health and medicine, researchers are only beginning to use these tools to explore the clinical characteristics and predictive factors of patients with COVID-19.

**Objective:**

Our primary objectives are to describe the clinical characteristics and determine the factors that predict intensive care unit (ICU) admission of patients with COVID-19. Determining these factors using a well-defined population can increase our understanding of the real-world epidemiology of the disease.

**Methods:**

We used a combination of classic epidemiological methods, natural language processing (NLP), and machine learning (for predictive modeling) to analyze the electronic health records (EHRs) of patients with COVID-19. We explored the unstructured free text in the EHRs within the Servicio de Salud de Castilla-La Mancha (SESCAM) Health Care Network (Castilla-La Mancha, Spain) from the entire population with available EHRs (1,364,924 patients) from January 1 to March 29, 2020. We extracted related clinical information regarding diagnosis, progression, and outcome for all COVID-19 cases.

**Results:**

A total of 10,504 patients with a clinical or polymerase chain reaction–confirmed diagnosis of COVID-19 were identified; 5519 (52.5%) were male, with a mean age of 58.2 years (SD 19.7). Upon admission, the most common symptoms were cough, fever, and dyspnea; however, all three symptoms occurred in fewer than half of the cases. Overall, 6.1% (83/1353) of hospitalized patients required ICU admission. Using a machine-learning, data-driven algorithm, we identified that a combination of age, fever, and tachypnea was the most parsimonious predictor of ICU admission; patients younger than 56 years, without tachypnea, and temperature <39 degrees Celsius (or >39 ºC without respiratory crackles) were not admitted to the ICU. In contrast, patients with COVID-19 aged 40 to 79 years were likely to be admitted to the ICU if they had tachypnea and delayed their visit to the emergency department after being seen in primary care.

**Conclusions:**

Our results show that a combination of easily obtainable clinical variables (age, fever, and tachypnea with or without respiratory crackles) predicts whether patients with COVID-19 will require ICU admission.

## Introduction

The unprecedented global spread of SARS-CoV-2, the virus that causes COVID-19, requires innovative approaches that deliver real-time results [1,2]. To date, big data analytics have been primarily used to assess SARS-CoV-2 transmission [3] and to indirectly estimate COVID-19 incidence using data from social media [4]. However, many factors involved in the onset and temporal distribution of the ongoing COVID-19 pandemic remain unknown. Similarly, both the individual and population burdens of COVID-19 are only beginning to be elucidated. Although big data analytics and artificial intelligence (AI) are widely used in the realms of health and medicine [5-7], researchers are only beginning to use these tools to explore the clinical characteristics and predictive factors of patients with COVID-19, including mortality [8-11].

Considering the unprecedented spread and severity of the ongoing COVID-19 outbreak, focus has been given to hospitals’ unmet needs, particularly their ICU requirements [8,9,12]. Indeed, health systems have been or currently are near collapse, and independent modelling efforts have aimed at forecasting a number of epidemiological estimators, including ICU use [13-15].

Previously, our team reported that a combination of big data analytics and machine learning techniques helped better determine the quality of diagnosis and treatment of chronic obstructive pulmonary disease (COPD) via an analysis of hospital electronic health records (EHRs) using natural language processing (NLP) and validated algorithms [16,17].

As part of the BigCOVIData study, our primary objectives are to describe the clinical characteristics and determine the factors that predict ICU admission of patients with COVID-19. Determining these factors using a well-defined population can increase our understanding of the real-world epidemiology of the disease. To achieve this aim, we used a combination of classic epidemiological methods [18], NLP, and machine learning (for predictive modeling) to analyze the clinical information contained in the EHRs of patients with COVID-19.

## Methods

The BigCOVIData study was conducted in compliance with legal and regulatory requirements and followed generally accepted research practices described in the International Council for Harmonisation of Technical Requirements for Pharmaceuticals for Human Use (ICH) Guideline for Good Clinical Practice, the latest edition of the Helsinki Declaration, the Guidelines for Good Pharmacoepidemiology Practices, and applicable local regulations. This study was classified as a “non–postauthorization study” by the Spanish Agency of Medicines and Health Products, and it was approved by the Research Ethics Committee at the University Hospital of Guadalajara (Spain). We and endorsed the STrengthening the Reporting of OBservational studies in Epidemiology (STROBE) guidance for reporting observational research [19].

### Study Design and Data Source

**Figure 1:**
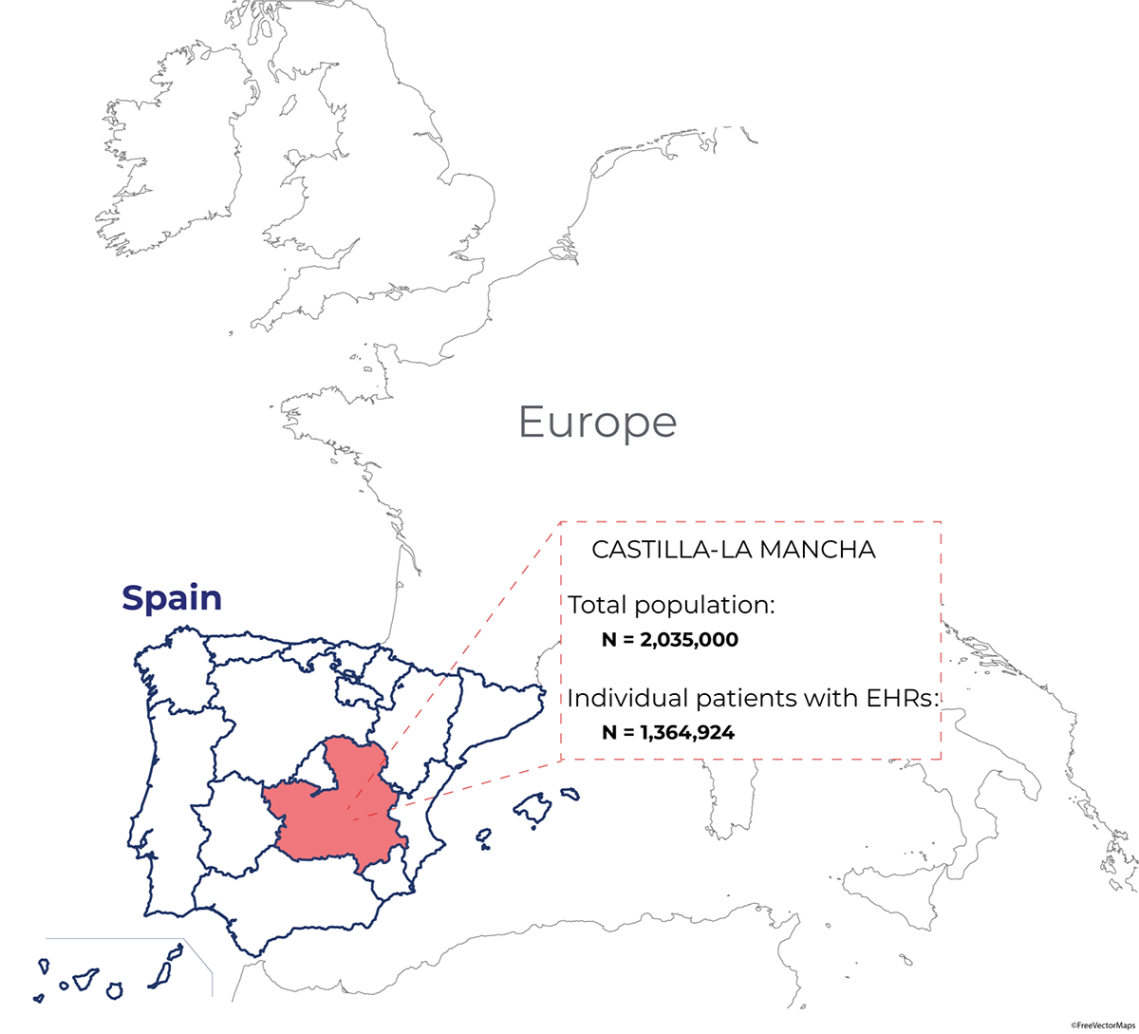
Map of the Castilla-La Mancha region (red) within the Spanish (blue line) and European territories. From a general source population of 2,035,000 inhabitants, we collected and analyzed the clinical information in the EHRs of 1,364,924 patients within the Servicio de Salud de Castilla-La Mancha (SESCAM) Health Care Network. EHR: electronic health record.

This was a multicenter, noninterventional, retrospective study using data captured in the EHRs of the participating hospitals within the Servicio de Salud de Castilla-La Mancha (SESCAM) Health Care Network in Castilla-La Mancha, Spain (Figure 1). Data captured in EHRs were collected from all available departments, including inpatient hospital, outpatient hospital, and emergency department (ED), for virtually all types of provided services in each participating hospital. The study period was January 1 to March 29, 2020.

The study database was fully anonymized in a structured format and contained no personal information from patients. Likewise, personal information was not accessed during either the application of automated and algorithmic methods (ie, NLP) or the conversion of unstructured data into the structured database. Importantly, given that clinical information was handled in an aggregate, anonymized, and irreversibly dissociated manner, patient consent regulations do not apply to the present study.

### Study Sample

The study sample included all patients in the source population who were diagnosed with COVID-19. Patients were identified on the basis of clinical diagnosis or microbiological test results. Clinical confirmation of COVID-19 cases was determined by observed symptomatology, imaging (mostly chest X-ray), and laboratory results, as captured in the unstructured, free-text information in the EHRs. Microbiological test result confirmation of COVID-19 cases involved reverse transcriptase–polymerase chain reaction (RT-PCR) or similar available tests. Our decision to consider cases confirmed both clinically and by RT-PCR was justified by the limited availability of routinely administered RT-PCR tests in the region during the study period and supported by recent discussions on the far-from-optimal sensitivity of RT-PCR for COVID-19 (ie, a single negative result from a single specimen cannot exclude the disease in suspected cases) [20,21]. Indeed, recent reports highlight the clinical validity and relatively high sensitivity of symptom- and imaging-based identification of patients with COVID-19, especially in early stages of the disease [20,22,23].

### EHRead

To meet the study objectives, we used EHRead [24], a technology developed by Savana that applies NLP, machine learning, and deep learning to analyze the unstructured free-text information written in millions of deidentified EHRs. This technology enables the extraction of information from all types of EHRs and subsequent normalization of the extracted clinical entities to a unique terminology. This process enables further analysis of a descriptive or predictive nature. Originally based on Systematized Nomenclature of Medicine Clinical Terms (SNOMED CT) terminology, our unique body of terminology comprises more than 400,000 medical concepts, acronyms, and laboratory parameters aggregated over the course of five years of free-text mining, targeting the most common diseases (eg, respiratory diseases, cardiovascular diseases, and diabetes).

Using a combination of regular expression rules and machine learning models, the terminology entities are detected in the unstructured text and later classified based on sections typically contained in EHRs, hospital services, and other clinical specifications. Importantly, each detected term is described in terms of negative, speculative, or affirmative clinical statements; this is achieved by using deep learning convolutional neural network classification methods that rely on word embeddings and context information (for a similar methodological approach, see [25]). The limitations of case-by-case detection are also overcome with a similar approach to ensure that the detected concepts are used within the appropriate context for the descriptive and predictive analysis.

For particular cases where extra specifications are required (ie, to differentiate COVID-19 cases from other mentions of the term related to fear of the disease or to potential contact), the detection output was manually reviewed in more than 5000 reports to avoid any possible ambiguity associated with free-text reporting. All NLP deep learning models used in this study were validated using the standard training/validation/testing approach; we used a 75/12/13 split ratio in the available annotated data (between 2000 and 3000 records, depending on the model) to ensure efficient generalization on unseen cases. For all developed models, we obtained F scores greater than 0.89.

### Data Analyses

All categorical variables (eg, comorbidities, symptoms) are shown in frequency tables, whereas continuous variables (eg, age) are described via summary tables that include the mean, SD, median, minimum and maximum, and quartiles of each variable. To test for possible statistically significant differences in the distribution of categorical variables between study groups (ie, male vs female, ICU admission vs no ICU admission), we used Yates-corrected chi-square tests. For continuous variables, mean differences were tested using *t* tests. Given our general population approach and our unusually large sample size, we were interested in exploring sex-related differences in patients with COVID-19; therefore, most results were stratified by sex [26]. All statistical inferences were performed at the 5% significance level using 2-sided tests or 2-sided CIs.

### Predictive Model

We developed a decision tree to classify patients with COVID-19 according to their risk of being admitted to the ICU. The two types of patients or *classes* considered in the model were therefore “admitted to the ICU” and “not admitted to the ICU.” The model maps the characteristics of the patients (the *variables*) to their class in the shape of a tree. From a clinical perspective, this model contemplates all patient variables upon admission; therefore, it is predictive from symptom debut until outcome. The tree is composed of nodes that branch to subsequent child nodes depending on the patient’s variables. The tree is built in such a way that each branch separates the two classes as much as possible. This separation is measured as the Shannon entropy, where a node with an entropy of zero indicates that the classification is perfect (either all or none of the patients were admitted to the ICU) and an entropy of one is the worst possible mix (50%/50%) [27].

### Model Training and Validation

The model was developed and tested on the available data from hospitalized patients who had or had not been admitted to the ICU; the latter were either discharged from the hospital or died in the course of the disease. This amounted to a total of 900 patients. We validated our algorithm by splitting our COVID-19 sample into a 70% training set and a 30% validation set. This means that the model was trained with 630 patients (582 who did not require intensive care vs 48 who did) and validated over the remaining 270 patients. Because the two classes were unbalanced (far fewer patients required ICU admission), we used the standard technique of oversampling the lower class to guarantee a balance of accuracy and recall (ie, the tradeoff between false positives vs false negatives). Further, we sought to replicate the results of this validation in an a posteriori sensitivity analysis, as per recent recommendations for predictive modeling in COVID-19 [28] and TRIPOD (Transparent Reporting of a multivariable prediction model for Individual Prognosis or Diagnosis) guidance [29]. For this second validation, we trained the model with data from the provinces of Ciudad Real and Guadalajara (38% of the study sample from Castilla-La Mancha), and we used an independent set with combined data set from the other three provinces, namely Toledo, Cuenca, and Albacete, for validation.

Additional details regarding the development and validation of the predictive algorithm are included in the Supplementary Methods in Multimedia Appendix 1. The workflow used for the generation of the predictive algorithm is summarized in Figure S1 in Multimedia Appendix 1.

## Results

**Figure 2:**
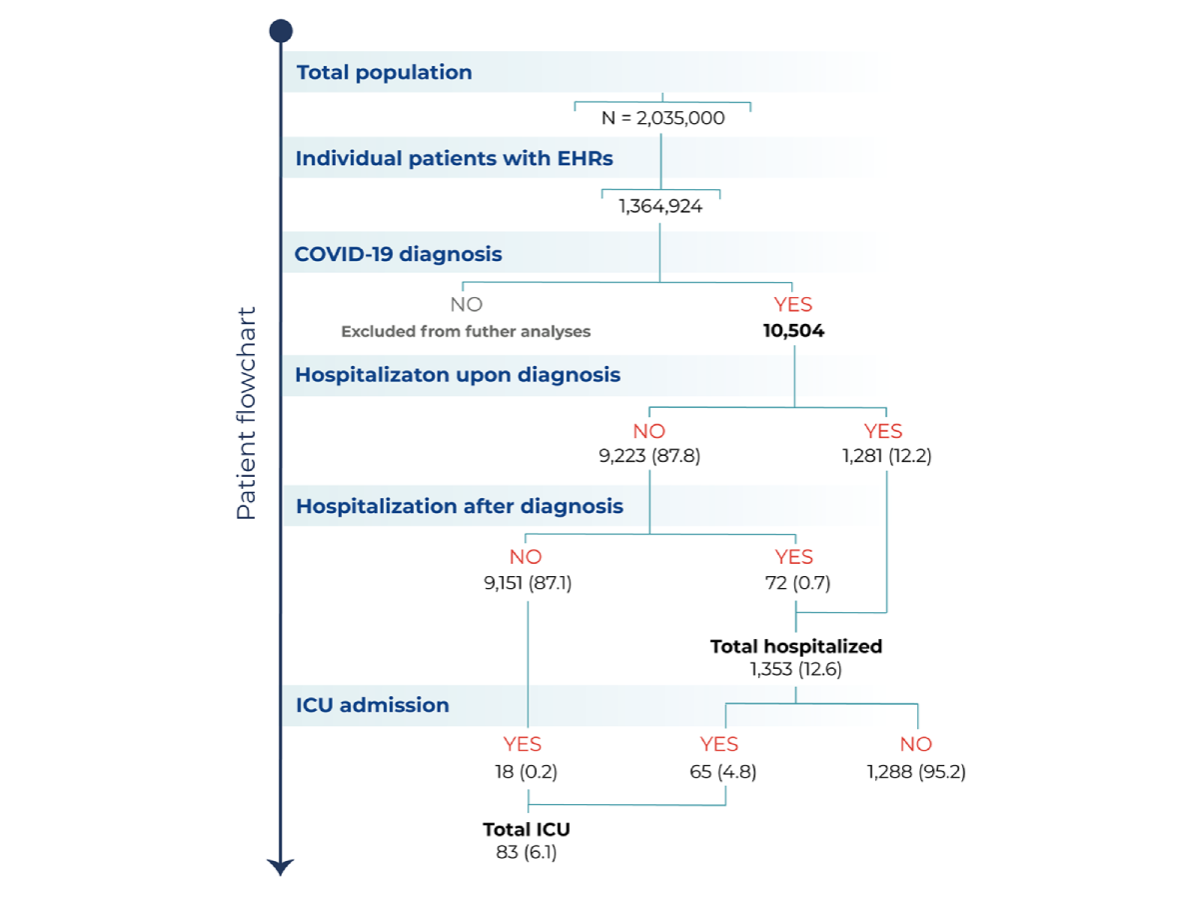
Patient flowchart depicting the total number of inhabitants in the source population, the number (%) of patients with available EHRs analyzed, the number of patients diagnosed with COVID-19, and of those, the number of hospitalizations and ICU admissions. EHR: electronic health record; ICU: intensive care unit.

From a source general population of 2,035,000 inhabitants, we used NLP and machine learning to analyze the clinical information contained in the EHRs of 1,364,924 anonymous patients (Figure 1). Among these, we identified a total of 10,504 patients diagnosed with COVID-19 (Figure 2). The flowchart of participation in the study up to hospital admission, ICU admission, or discharge is presented in Figure 2.

**Figure 3:**
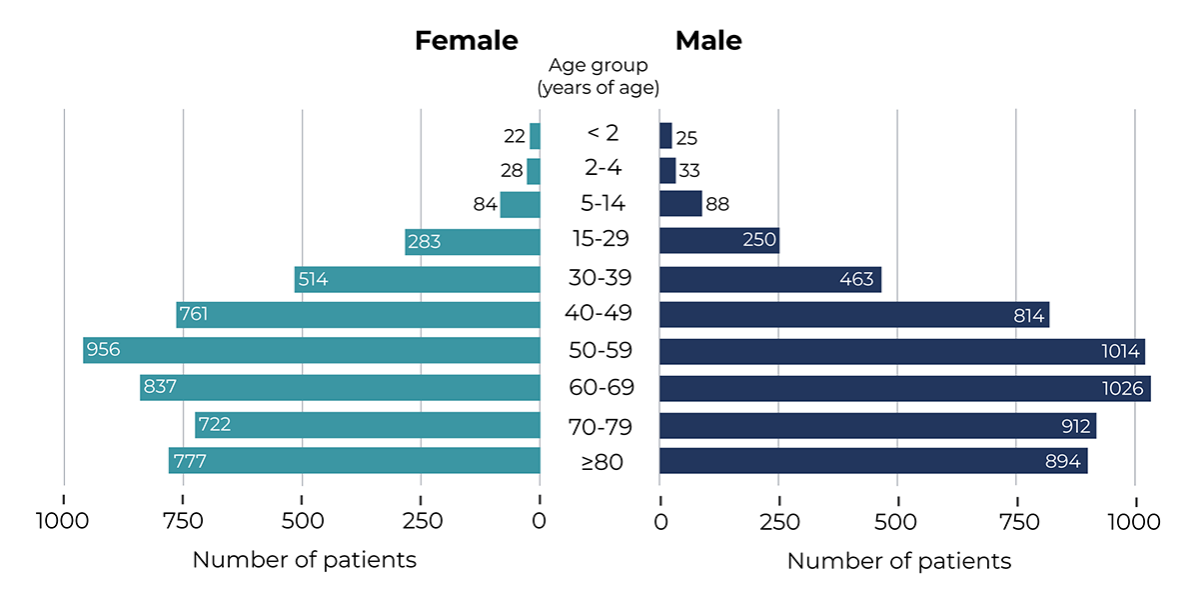
Age distribution of incident cases of COVID-19 in females (left) and males (right) in the study population for the period comprised between Jan 1, 2020 and March 29, 2020.

Of the patients with COVID-19, 52.5% (5519/10,504) were male, with a mean age of 58.2 years (SD 19.7) (Table 1). Most patients with COVID-19 were aged ≥50 years (Figure 3). Upon diagnosis, the most common symptoms reported were cough, fever, and dyspnea (Table 1); notably, less than half of patients presented with all three of these symptoms. Further, respiratory crackles, myalgia, and diarrhea were identified in ≥5% of cases, while other respiratory and nonrespiratory signs and symptoms were less common. Sex-dependent differences in sign and symptom frequencies upon diagnosis are shown in Table 1. Of note, we observed subtle increases in the frequency of diarrhea, myalgia, headache, chest pain, and anosmia in female patients with COVID-19, while male patients showed significant increases in fever, dyspnea, respiratory crackles, rhonchus, lymphopenia, and tachypnea (all *P*<.05).

**Table 1:** Baseline demographics and clinical data of the patients in the study upon diagnosis (N=10,504).

Characteristic			Female	Male	Total		*P* value^a^
Sex^b^, n (%)			4984 (47.4)	5519 (52.5)	10,504 (100)		N/A^c^
**Age (years)**						<.001	
	Mean (SD)		57.4 (20.0)	59.0 (19.5)	58.2 (19.7)		
	Median (minimum-maximum)		58.0 (0.0-100.0)	60.0 (0.0-102.0)	59.0 (0.0-102.0)		
	Q1-Q3		44.0-73.0	46.0-74.0	45.0-73.0		
**Signs and symptoms, n (%)**							
	Cough		2482 (49.8)	2760 (50.0)	5243 (49.9)		.85
	Fever		2120 (42.5)	2783 (50.4)	4904 (46.7)		<.001
	Dyspnea		1476 (29.6)	1818 (32.9)	3294 (31.4)		<.001
	Respiratory crackles		849 (17.0)	1085 (19.7)	1934 (18.4)		<.001
	Diarrhea		556 (11.2)	543 (9.8)	1099 (10.5)		.03
	Myalgia		467 (9.4)	451 (8.2)	919 (8.7)		.03
	Headache		462 (9.3)	302 (5.5)	764 (7.3)		<.001
	Rhonchus		279 (5.6)	414 (7.5)	693 (6.6)		<.001
	Chest pain		287 (5.8)	267 (4.8)	554 (5.3)		.04
	Lymphopenia		196 (3.9)	346 (6.3)	542 (5.2)		<.001
	Wheezing		194 (3.9)	195 (3.5)	389 (3.7)		.36
	Tachypnea		135 (2.7)	203 (3.7)	338 (3.2)		.006
	Anosmia		166 (3.3)	134 (2.4)	300 (2.9)		.007
	Sore throat		69 (1.4)	57 (1.0)	127 (1.2)		.12
	Ageusia		33 (0.7)	32 (0.6)	65 (0.6)		.68
	Dysphagia		19 (0.4)	28 (0.5)	47 (0.4)		.41
	Neuralgia		19 (0.4)	22 (0.4)	41 (0.4)		>.99
	Splenomegaly		8 (0.2)	14 (0.3)	22 (0.2)		.41
	Hepatomegaly		2 (0.0)	6 (0.1)	8 (0.1)		.36
**Comorbidities^d^, n (%)**							
	**Cardiovascular disease**		2253 (45.2)	2805 (50.8)	5058 (48.2)		<.001
		Hypertension	1552 (31.1)	1975 (35.8)	3527 (33.6)		<.001
		Ischemic stroke	91 (1.8)	163 (3.0)	254 (2.4)		<.001
	**Heart disease**		1100 (22.1)	1539 (27.9)	2639 (25.1)		<.001
		Ischemic heart disease	152 (3.0)	475 (8.6)	627 (6.0)		<.001
		Heart failure	243 (4.9)	309 (5.6)	552 (5.3)		.11
	Diabetes		689 (13.8)	957 (17.3)	1646 (15.7)		<.001
	Obesity		479 (9.6)	457 (8.3)	936 (8.9)		.02
	**Renal dysfunction**		271 (5.4)	493 (8.9)	764 (7.3)		<.001
		CKD^e^	171 (3.4)	323 (5.9)	494 (4.7)		<.001
	Depression		484 (9.7)	219 (4.0)	703 (6.7)		<.001
	**Chronic respiratory disease**		242 (4.9)	646 (11.7)	888 (8.5)		<.001
		Asthma	496 (10.0)	263 (4.8)	759 (7.2)		<.001
		COPD^f^	126 (2.5)	549 (9.9)	675 (6.4)		<.001
		OSA^g^	69 (1.4)	143 (2.6)	212 (2.0)		<.001
		Bronchiectasis	42 (0.8)	87 (1.6)	129 (1.2)		<.001
	**Chronic liver disease**		36 (0.7)	75 (1.4)	111 (1.1)		.002
		Cirrhosis	16 (0.3)	35 (0.6)	51 (0.5)		.03
	HIV		12 (0.2)	22 (0.4)	34 (0.3)		.21

^a^*P* values from Yates-corrected chi-square test on percentage difference of female vs male COVID-19 patients. All tests were performed individually for each variable (sign, symptom, or comorbidity, where applicable). For numerical values (ie, age), *t* tests of difference between means were used.

^b^The sex of one patient was listed as Unknown.

^c^N/A: not applicable.

^d^List of medical conditions according to Systematized Nomenclature of Medicine Clinical Terms terminology.

^e^CKD: chronic kidney disease.

^f^COPD: chronic obstructive pulmonary disease.

^g^OSA: obstructive sleep apnea.

Similarly, the most frequent comorbidities among the 10,504 patients were cardiovascular disease (n=5058, 48.2%), mainly arterial hypertension (n=3527, 33.6%); heart disease (n=2639, 25.1%); and diabetes (n=1646, 15.7%) (Table 1). Regarding respiratory diseases, COPD was present in 6.4% (675), asthma in 7.2% (759), obstructive sleep apnea (OSA) in 2% (212), and bronchiectasis in 1.2% (129) of the 10,504 patients. Sex-dependent differences in comorbidities upon diagnosis are also shown in Table 1; except for asthma, the frequency of all comorbidities was significantly higher in male than in female patients with COVID-19 (all *P*<.05).

Next, we explored whether the distribution of comorbidities and signs and symptoms captured in the patients’ EHRs upon diagnosis differed between patients with COVID-19 who were and were not admitted to the ICU (Table 2). Regarding comorbidities, diabetes, obesity, cardiovascular disease (mainly hypertension), heart disease (mainly ischemic heart disease), and renal dysfunction were more common among patients who were admitted to the ICU (all *P*<.01). As for signs and symptoms, cough, fever, dyspnea, respiratory crackles, diarrhea, tachypnea, lymphopenia, and rhonchus were more frequent among ICU patients (all *P*<.05). Interestingly, respiratory diseases were not more frequent among patients who were admitted to the ICU (Table 2).

**Table 2:** Associations of signs and symptoms and comorbidities with ICU admission upon diagnosis in patients with COVID-19 (N=10,504).

Variable			Not admitted to ICU^a^ (n=10,421), n (%)	Admitted to ICU (n=83), n (%)	*P* value^b^
**Signs and symptoms**					
	Cough		5181 (49.7)	62 (74.7)	<.001
	Fever		4849 (46.5)	55 (66.3)	<.001
	Dyspnea		3246 (31.1)	48 (57.8)	<.001
	Respiratory crackles		1904 (18.3)	30 (36.1)	<.001
	Myalgia		908 (8.7)	11 (13.3)	.21
	Diarrhea		1084 (10.4)	15 (18.1)	.04
	Dysphagia		47 (0.5)	0 (0)	>.99
	Wheezing		383 (3.7)	6 (7.2)	.16
	Tachypnea		311 (3)	27 (32.5)	<.001
	Chest pain		546 (5.2)	8 (9.6)	.12
	Lymphopenia		524 (5)	18 (21.7)	<.001
	Headache		757 (7.3)	7 (8.4)	.84
	Rhonchus		676 (6.5)	17 (20.5)	<.001
	Hepatomegaly		8 (0.1)	0 (0)	>.99
	Anosmia		297 (2.9)	3 (3.6)	.93
	Ageusia		65 (0.6)	0 (0)	.98
	Neuralgia		41 (0.4)	0 (0)	1
	Sore throat		126 (1.2)	1 (1.2)	1
	Splenomegaly		21 (0.2)	1 (1.2)	.43
**Comorbidities^c^**					
	Diabetes		1613 (15.5)	33 (39.8)	<.001
	Obesity		917(8.8)	19 (22.9)	<.001
	**Chronic respiratory disease**		883 (8.5)	5 (6)	.55
		COPD^d^	673 (6.5)	2 (2.4)	.20
		Asthma	750 (7.2)	9 (10.8)	.29
		OSA^e^	211 (2)	1 (1.2)	.89
		Bronchiectasis	129 (1.2)	0 (0)	.60
	**Cardiovascular disease**		4998 (48)	60 (72.3)	<.001
		Hypertension	3487 (33.5)	40 (48.2)	.007
		Ischemic stroke	253 (2.4)	1 (1.2)	.72
	**Heart disease**		2604 (25)	35 (42.2)	<.001
		Ischemic heart disease	616 (5.9)	11 (13.3)	.01
		Heart failure	548 (5.3)	4 (4.8)	>.99
	**Renal dysfunction**		748 (7.2)	16 (19.3)	<.001
		CKD^f^	488 (4.7)	6 (7.2)	.41
	**Chronic liver disease**		109 (1)	2 (2.4)	.50
		Cirrhosis	51 (0.5)	0 (0)	>.99
	Depression		699 (6.7)	4 (4.8)	.64
	HIV		33 (0.3)	1 (1.2)	.65

^a^ICU: intensive care unit.

^b^*P* values from Yates-corrected chi-square tests of differences between percentages of patients in either outcome group. All tests were performed individually for each variable (sign, symptom, or comorbidity, where applicable).

^c^List of medical conditions according to Systematized Nomenclature of Medicine Clinical Terms terminology.

^d^COPD: chronic obstructive pulmonary disease.

^e^OSA: obstructive sleep apnea.

^f^CKD: chronic kidney disease.

**Figure 4:**
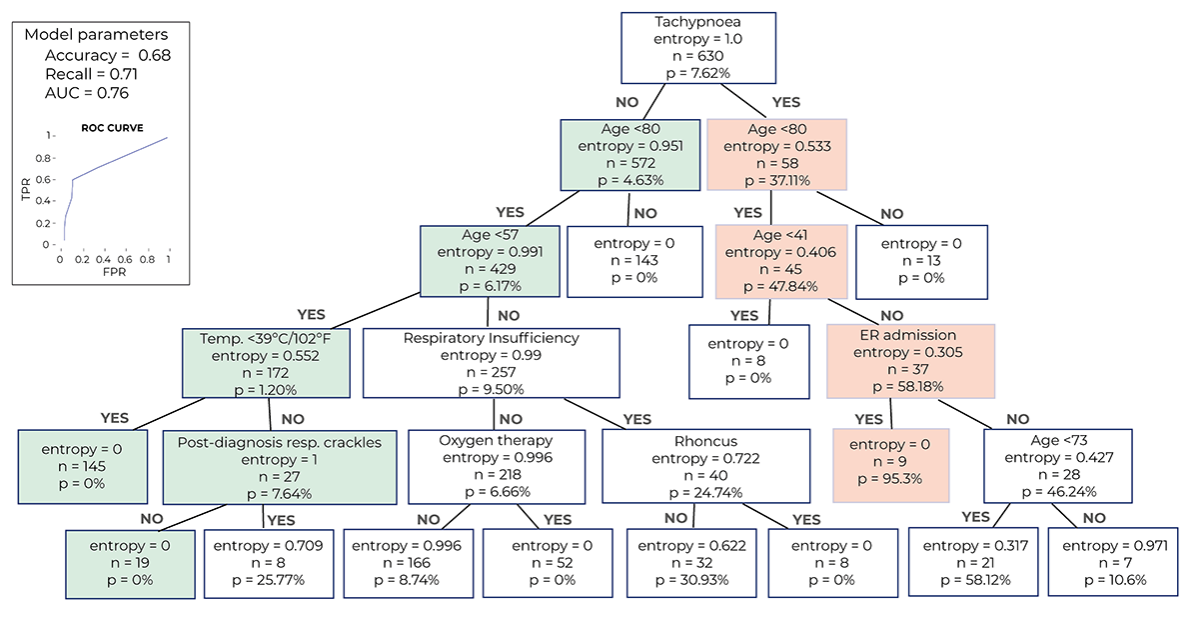
Decision tree of relevant clinical variables for the prediction of ICU admission in patients with COVID-19. The combination of three easily available clinical variables, namely age, temperature, and respiratory frequency, was the most parsimonious predictor of ICU admission among COVID-19 patients. The number of patients, probability (p) of ICU admission predicted by the model, and level of entropy (a measure indicating how mixed or pure the classification is, where 0 indicates optimal separation of classes) are indicated in each box. The green pathway indicates that patients with no tachypnea, age <56 years, and temperature <39 ºC (OR >39 ºC without respiratory crackles) did not require ICU admission. In contrast, the red pathway indicates that patients aged 40-79 years, who presented with tachypnea, and who delayed their visit to the emergency department after being seen in primary care were likely to be admitted in the ICU. For this model, we obtained accuracy, recall, and AUC values of 0.68, 0.71, and 0.76, respectively (top right panel). AUC: area under the curve; FPR: false positive rate; resp.: respiratory; ROC: receiver operating characteristic; TPR: true positive rate.

Finally, by using a machine-learning, data-driven algorithm, we identified that a combination of three easily available clinical variables, namely age, temperature, and respiratory frequency, was the most parsimonious predictor of ICU admission among patients with COVID-19 (Figure 4). For this model, age and temperature were captured as continuous variables, whereas tachypnea (yes/no) was defined as a respiratory frequency of more than 20 breaths per minute. With accuracy, recall, and area under the curve (AUC) values of 0.68, 0.71, and 0.76, respectively, the presented model reached optimal balance in terms of positive and negative predictive values for ICU admission. On the one hand, patients younger than 56 years, without tachypnea, and with temperature <39 ºC (entropy=0, n=145) or >39 ºC without respiratory crackles (entropy=0, n=18) were not admitted to the ICU. On the other hand, patients with COVID-19 aged 40 to 70 years were likely to be admitted in the ICU if they presented with tachypnea and delayed their visit to the ED after being seen in primary care (entropy=0, n=104). As stated in the Methods section, we performed an additional sensitivity analysis with different data sets to further validate the results of our predictive model. The independent data set of two provinces (Ciudad Real and Guadalajara, including a total of 753,408 individual patients, or 38% of the entire study sample from Castilla-La Mancha; Figure 1 and Supplemental Table S1 in Multimedia Appendix 1), was used to retrain our algorithm to identify ICU admission at onset; validation was performed in the remaining three provinces. As shown in Supplemental Figure S2 in Multimedia Appendix 1, the new decision tree identified the same relevant clinical variables, that is age, tachypnea, temperature, and respiratory crackles/rhonchus, with similar (but not identical) thresholds in some variables. This additional model achieved accuracy, recall, and AUC values of 0.85, 0.57, and 0.84, respectively, providing additional proof of validity for our main findings.

## Discussion

### Principal Findings

By accessing the clinical information of more than 10,000 anonymous patients with COVID-19 (a number that largely surpasses samples included in recent reports about the disease [30,31]), we were able to describe their demographic and clinical characteristics, their clinical journey, and the statistical relationship between the most common symptoms and comorbidities on admission, and COVID-19 prognosis (ie, ICU admission). There were subtle differences in clinical symptoms at onset by sex, while all comorbidities (except asthma) were significantly more frequent in male than female patients with COVID-19. The variables identified in our ICU admission model (ie, age, temperature, and tachypnea) are clinically relevant, as they are readily available and easily observable in everyday practice for patients with COVID-19. Although tachypnea is not an exclusive manifestation of COVID-19 and can be present in patients suffering from other respiratory diseases (ie, pneumonia), our model suggests that this sign (in combination with age and temperature) is a more reliable predictor of ICU use than other common symptoms and signs, such as cough, dyspnea, or respiratory crackles.

The reported sex-dependent differences in the symptomatology of COVID-19 at onset have been further confirmed by our group using similar methods [32] and should be interpreted in light of data suggesting that female teenagers and young adult women are significantly more affected by the disease than their male counterparts [32]. In this regard, our results warrant further investigations aimed at closing the gender gap in the ongoing pandemic [33].

Given that the stability and capacity of ICUs worldwide is threatened by the rapid spread of COVID-19, the identification of individual factors that predict ICU admission may not only improve patient management but also optimize health care resource use and planning. Thus, recent studies using big data and machine learning have explored the prognostic factors of the disease, including ICU transfer, discharge, and mortality [8-11]. In line with our results, respiratory rate has also been identified as an important predictor of ICU transfer in patients with COVID-19 [9].

If further applied to other national and international health care networks, the tools and methodology presented in this study can potentially characterize and predict the prognosis of COVID-19 in a timely and unprecedented manner. As demonstrated in recent studies [34,35], there may be value in the application of AI to the current COVID-19 pandemic, not only to predict outbreaks [36] or read chest computed tomography scans [37] but also to elucidate the clinical onset and natural history of COVID-19 almost in real time. Indeed, classical methods would require months of questionnaire-based data collection and questionnaire validation along with multiple Ethics Board approvals and other practical hurdles; these steps can be avoided with our current approach.

In the race against COVID-19 [38], where the goal is to curb the pandemic, it is imperative to leverage big data and intelligent analytics for the betterment of public health. However, it is of the utmost importance not to neglect privacy and public trust, to apply best practices, and to maintain responsible standards for data collection and data processing on a global scale [39].

### Strengths and Limitations

To our knowledge, this is one of the first attempts to combine NLP and machine learning to access and analyze unstructured, free-text real-world data from EHRs in a large series of patients with COVID-19. Although recent studies have used machine learning to predict ICU admission in patients with COVID-19 [9], our approach takes this methodology one step further by applying NLP to exclusively analyze unstructured information. Indeed, our state-ot-the-art methodology enabled rapid analysis of the unstructured free-text narratives in the EHRs of 1 million patients from the general population of the region of Castilla-La Mancha (Spain).

Our methodology combined modules for sentence segmentation, tokenization, text normalization, acronym disambiguation, negation detection, and a multidimensional ranking scheme; the latter involved linguistic knowledge, statistical evidence, and continuous vector representations of words and documents learned via shallow neural networks. When applied to EHRs, NLP enables both access to longitudinal health records for *all* patients in the target population and the implementation of exploratory analysis to clarify associations between variables that have remained undetected with traditional research methods. By considering all possible patients with the target disease, the information and analyses used here (ie, real-world data and free-scale statistics) remained unbiased by the research question or the observers. Unlike classical statistical methods (eg, logistic regression), the main advantage associated with the use of machine learning in this context is that it enables the automatic detection of meaningful relationships between variables. For instance, if a given symptom (ie, fever) is only relevant for certain patients (ie, older than 50 years), techniques such as the classification trees used here are suitable to uncover this relationship. In this context, although the total number of patients who required ICU use in the training set was somewhat low (48 patients), the number of variables considered in the model was also very limited. In addition, the inclusion of a validation stage reduces the likelihood of overfitting. Ultimately, the use of classification trees in this study (as opposed to other models, such as artificial neural networks) is especially appropriate in the clinical context because they are easily interpretable.

Regarding the geographical location of our participating hospital sites, it is worth mentioning that with a total of 1,364,924 patients from the region of Castilla-La Mancha (SESCAM Health Care Network), our sample is representative of the Spanish population. Spain is among the countries that have been most affected by the pandemic in terms of both total cases and mortality rates [40,41]; the Castilla-La Mancha region in particular is the third most affected region in the country, just behind Madrid and Catalonia. For this reason, we anticipate that the clinical conclusions drawn here will be relevant for clinicians worldwide. Of note, the ICU capacity in the region during the study period had not yet been compromised, which protects against possible bias in our training data (all patients requiring intensive care were indeed admitted to the ICU).

The results and conclusions of the present study should be interpreted in light of the following limitations. First, we did not distinguish COVID-19 cases confirmed by laboratory results (ie, RT-PCR) from those exclusively diagnosed through clinical observation (ie, symptomatology, imaging and laboratory results). However, it should be noted that PCR and other rapid laboratory tests for the detection of SARS-CoV-2 were not routinely administered in Spain during the study period. In addition, this decision is supported by recent discussions on the clinical validity and relatively high sensitivity of symptom- and imaging-based identification of patients with COVID-19, especially in early stages of the disease [20,22,23]. Second, independent replications by different research groups in larger patient sets are needed to further support the clinical validity of our results.

Finally, future reports from the BigCOVIData study may incorporate laboratory results and treatments and may contextualize the results presented here in a larger clinical picture [28].

### Conclusion

In this study, we found that in the largest series of patients with COVID-19 attended during the first three months of the pandemic in Spain, 6.1% of all hospitalized patients (83/1353) required ICU admission. We also found that a combination of easily obtained clinical variables, namely age, fever, and tachypnea, predicts whether patients with COVID-19 will require ICU admission.

## Appendix

Multimedia Appendix 1 Supplementary Methods/Supplementary Figures and Tables.

## Abbreviations

AI: artificial intelligence
AUC: area under the curve
COPD: chronic obstructive pulmonary disease
ED: emergency department
EHR: electronic health record
ICH: International Council for Harmonisation of Technical Requirements for Pharmaceuticals for Human Use
ICU: intensive care unit
NLP: natural language processing
OSA: obstructive sleep apnea
RT-PCR: reverse transcriptase–polymerase chain reaction
SESCAM: Servicio de Salud de Castilla-La Mancha
SNOMED CT: Systematized Nomenclature of Medicine Clinical Terms
STROBE: STrengthening the Reporting of OBservational studies in Epidemiology
TRIPOD: Transparent Reporting of a multivariable prediction model for Individual Prognosis or Diagnosis
