# Supplementary material for: Clinical Characteristics and Prognostic Factors for Intensive Care Unit Admission of Patients With COVID-19: Retrospective Study Using Machine Learning and Natural Language Processing
Source: J Med Internet Res. 2020 Oct 28;22(10):e21801. doi: 10.2196/21801 (PMC7595750; doi:10.2196/21801)
Supplement: Multimedia Appendix 1 [file jmir_v22i10e21801_app1.doc]

**SUPPLEMENTARY INFORMATION**

**CLINICAL CHARACTERISTICS AND PROGNOSTIC FACTORS FOR ICU ADMISSION OF PATIENTS WITH COVID-19: A RETROSPECTIVE STUDY USING MACHINE LEARNING AND NATURAL LANGUAGE PROCESSING**

**SUPPLEMENTARY METHODS**

Developing a predictive algorithm for ICU admission in patients with COVID-19

The goal of our predictive algorithm was to classify COVID-19 patients according to their risk of being admitted to the ICU. Thus, the two types of patients or *classes* we considered were "patient admitted to the ICU" and "patient not admitted to the ICU". The model maps the characteristics of patients (the *variables*) to their class in the shape of a tree. The following sections include additional details regarding the development of the model, from data organization to interpretation of results. The pipeline used for the generation of the predictive model presented here include the following steps (Figure S1):

*Building the ‘master’ table*. Data are first organized in the so-called ‘master’ table. This two-dimensional table consists of a subset of the aggregated study database that only contains the clinical information relative to the study objectives, in turn specified by medical experts prior to NLP processing. In this table, each row represents a single, anonymous patient; columns contain the different variables included in the study.

*Cleansing and optimizing the ‘master’ table*. Because not all variables are detected or read in all patients included in the master table, most columns (i.e., study variables) will contain a significant number of empty cells. These missing values need to be addressed and dealt with before the predictive algorithm can be trained. In the data cleansing process, we distinguish three types of variables, namely binary variables (yes/no presence of the variable or term), numeric variables (e.g., laboratory values), and multi-class variable (i.e., stage of cancer). The procedures used for the imputation of missing data vary across variable types:

- 1. For binary variables, missing data are treated as true ‘0’ values (i.e., no apparition of the variable)
  2. For numeric variables, missing data are filled with the median value for existing data
  3. For multi-class variables, missing data are filled with the median value for existing data

*Definition of dependent and independent variables.* In this step, the variable that the model aims to predict (i.e., dependent variable: ICU admission) is removed from the list of potential predictors (i.e., independent variables: age, temperature, symptoms, etc.).

*Feature selection algorithm (dimensionality reduction)*. To guarantee that the output of the model is easily interpretable from a clinical standpoint, the optimal number of independent variables (or features) must be lower than 10. To obtain the right number of features, random forest feature importance was used.

*Prediction algorithm*. The final stage in the generation of the algorithm involves the following procedures:

1. Train and split of the data. To train and validate the models, the dataset is separated in a 70/30 training/validation split. This means that 70% of the data is used to train and fine-tune the weights of the variables, and the remaining 30% is used to validate or test the performance of the model.
2. Data augmentation. Due to the imbalance nature of the datasets, standard upsampling techniques were used to balance the number of positive and negative cases. This step is performed prior to training the model.
3. Output. Two types of models are trained based on their clinical interpretability, namely decision trees or logistic regression. Both algorithms rely on a set of parameters which are optimized (i.e., hyper-parameter optimization) to reach high performance without adding too much complexity. The most meaningful model is then kept and interpreted by medical experts in order to produce the final predictive algorithm (in the case of the present paper, the decision tree classifying patients according to their risk to be admitted in the ICU).

*Interpretation of results*. Finally, the predictive models are assessed in terms of AUC (Area Under the Curve), recall, and accuracy (see main text for performance metrics associated with our prediction model for ICU admission in COVID-19 patients).

*Additional model validation*. To further validate the results obtained, predictive models should be validated in *a posteriori* sensitivity analysis. For the presented model predicting ICU admission, we opted for a geographical split of the target population (see main text for details).

**SUPPLEMENTARY FIGURES**


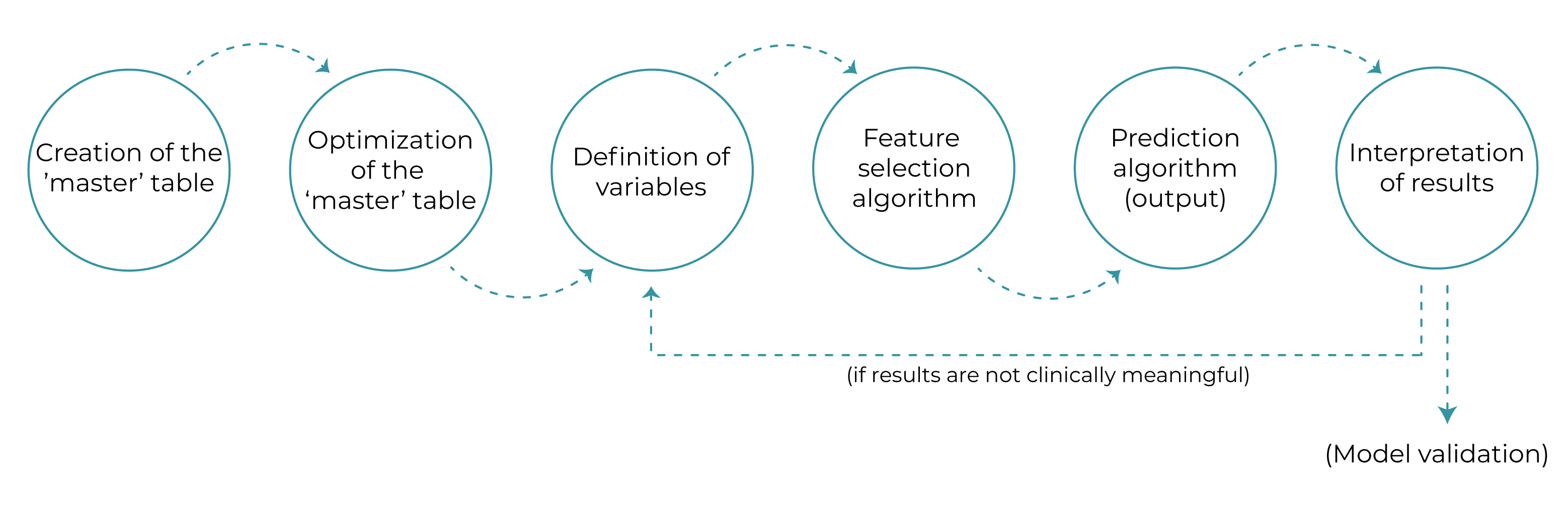


**Figure S1. Flowchart depicting the workflow for the predictive algorithm.** The construction of the predictive model starts with the creation and optimization of a ‘master’ table. This table represents a subset of the aggregated study database, and only contains the clinical information relevant to pursue the study aims. Once the master table has been created and cleaned up, the next step is to define the dependent (i.e., what to predict) and independent (i.e., predictors) variables to include in the model. Next, feature selection algorithms are implemented to reduce the dimensionality of variables included in the model. The generation of the predictive algorithm (output) involves splitting the data for training and validation. Models are trained based on their clinical interpretability, which is assessed in terms of AUC, recall, and accuracy. Finally, additional validation with external datasets is advisable (see main text for details).


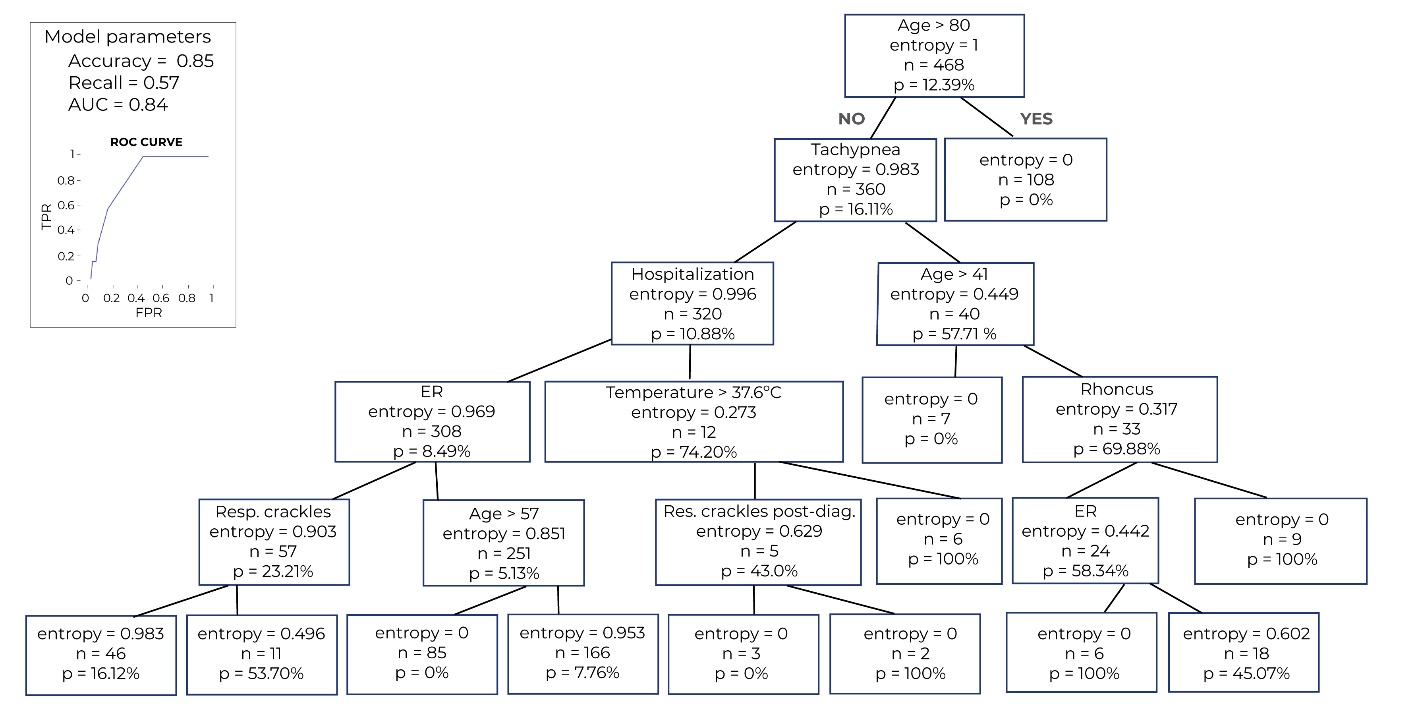


**Figure S2. A posteriori, sensitivity analysis of the decision tree of relevant clinical variables for the prediction of ICU admission in COVID-19 patients** The number of patients, probability (p) of ICU admission predicted by the model, and level of entropy (a measure indicating how mixed or pure the classification is, where 0 indicates optimal separation of classes) are indicated in each box. Among all considered variables, this additional sensitivity analysis reidentified age, tachypnea, temperature, and respiratory crackles as key factors to determine whether a patient COVID-19 is admitted to the ICU (Figure 4). Of note, the thresholds associated with each variable were similar but not identical in all instances. For this model, we obtained accuracy, recall, and AUC values of 0.85, 0.57, and 0.84, respectively (top right panel).

**SUPPLEMENTARY TABLES**

**Table S1**. Population distribution in Castilla-La Mancha and its five subregions.

| **Province** | **Population** | **%*** | **Patients /1,000 inhabitants** |
| --- | --- | --- | --- |
| Toledo | 687,391 | 34% | 3.51 |
| Ciudad Real | 499,100 | 25% | 6.62 |
| Guadalajara | 254,308 | 13% | 4.75 |
| Cuenca | 197,222 | 10% | 2.88 |
| Albacete | 388,786 | 19% | 8.00 |
| **Total** | 2,026,807 |  |  |

*Percentage of inhabitants relative to the total population of Castilla La-Mancha
